# A Visit to the Hospitals of Paris and to Pasteur’s Laboratory

**Published:** 1886-11

**Authors:** Charles Warrington Earle

**Affiliations:** 535 Washington Boulevard


### ABSTRACT OF A REPORT MADE OF A VISIT TO THE HOSPITALS OF PARIS AND PASTEUR'S LABORATORY. *By* CHARLES WARRINGTON EARLE, M.D.

[Read before the Chicago Medical Society, October 4, 1886.]

After my arrival in Paris, the Hospital Lariboisière was among the first that I visited. It is a large structure, containing 875 beds, and is situated in the northeastern part of the city. It was constructed in 1854, entirely of stone and brick. It has several pavilions and squares as well as gardens and chapels within its walls. The building is of historic interest in many respects, and still bears the scars of German cannon balls received during the late war. The obstetrical department is divided into an inside and outside service, with about thirty-five hundred confinements in both. Midwives do the work on the outside, and send for the hospital staff if assistance is needed. The severe cases needing operation are brought into the hospital and kept there until they have recovered. Every morning a large number of patients are brought in by the midwives to be examined by the resident physicians; if any complication is found, they are retained in the hospital. The wards are kept aseptic in every respect. Carbolic acid is distributed in dishes, and in many cases a spray of the same substance is used. The beds are all of iron, and are washed thoroughly from time to time with a solution of corrosive sublimate. The floors are of a stone and plaster composition, and the walls are roughly finished with not the faintest attempt at ornamentation. The antiseptic precautions are not equal to those taken in the hospitals of Germany and Italy, but they are in advance of those in England.

The favorite antiseptic is the biniodide of mercury in a strength of one part to two thousand of water. Pinard is the obstetrician, and his methods are peculiarly French. They dislike to use any method or instrument devised by a German. Tarnier's forceps, with certain recent modifications, by which they can be more easily rendered aseptic, are used, and the basotribe, a combination of the perforator and cranioclast has recently been added. When a diagnosis of breech presentation can be made early, before the escape of the amniotic fluid, version is performed. The general result of this treatment has not as yet been announced. I am, however, in possession of some facts which would make it appear that it is not the best procedure. In cases of contracted pelvis, labor is induced between the sixth and seventh months, and the prematurely delivered child is placed in the incubator, its life being saved as a rule. In Germany, Austria, and probably Italy, these cases would have some form of abdominal section performed upon them. The contrivances for keeping up the body-heat of prematurely born infants were very interesting to me. I did not see Credé's apparatus, which consists of a copper tube with double walls so arranged that hot water can be introduced, and a high temperature maintained, but I did see Tarnier's incubators in operation. The principle upon which they are constructed is very simple, and it seems to me that we should be in position to utilize it. They consist of a square box so made as to prevent the loss of heat, the top of which is covered with glass. In the upper half is placed a thermometer and a little basket of soft clothing for the child. Under the basket is an arrangement for the introduction of hot air, which may be supplied either from the heating apparatus of the building, a gas jet, or hot water in bottles. For the youngest children the temperature is kept from 100° to 105° F.; for those between seven and eight months from 85° to 100° F. Where possible, the children are always fed with breast-milk and are not handled or exposed more than is absolutely necessary. A sloughing uterine tumor or other case in which blood-poisoning may happen, is treated by constant irrigation; water, either pure or slightly medicated, is passed into the cavity for days and often weeks, thus effectually washing away all putrid matter and guarding against infection.

I was shown an instrument recently devised by Tarnier for inducing labour. It was an ordinary catheter upon the end of which was a little bulb with exceedingly thin walls. The instrument is introduced into the cavity of the uterus, the bulb distended with water and allowed to remain until uterine action begins.

The Maison de Accouchements is Tarnier's own hospital. Formerly it was a Catholic institution, and as far as the building is concerned it is not at all in accord with recent ideas of what a hospital should be. Everything possible, however, is done to make the place aseptic. Every bed is isolated in some degree, and furnished with its own irrigator, the permanganate of potash being the drug used. Carbolic acid is sprayed in the wards at all times. The bedsteads and chairs are all iron. The floor is of stone, and the ventilation is by means of fireplaces. Tarnier has just completed a small ward from plans of his own which, in all probability, is the most non-absorbing building which has ever been erected. It is made of brick and mortar and stone, the roof of the porches is made of glass, the window-frames are all iron, and as far as

I could see not a particle of wood entered into the construction of the building. It is only 24x45 feet, and so arranged that one attendant upon each floor can look into the four wards from a shaft which is constructed in the center. This is to economize help and give to each patient constant watchfulness and care.

The Hospital Bichât is situated directly upon the fortifications. It was built for barracks, and has only recently been converted into a hospital. Terrier is the surgeon. The building is of stone with a perfectly hard and non-absorbing material for floors. The carbolic acid spray is used, and a very great amount of it is used in each surgical dressing. Operations for hernia seem to be a kind of specialty here, though many laparotomies are performed. It is in this hospital that Pean extirpates the uterus, sometimes without the use of a single ligature. He has had constructed for him, long hæmostatic forceps with different shapes and curves, and if an artery is cut high up, which it would be difficult to ligate, it is at once seized with one of these forceps, the instrument left in place for one or two days, when it sloughs away.

The Hospital Tenon is another institution which I visited. It is very large, is kept scrupulously clean and has an obliging staff of attendants who delight in showing one over the building.

Among those who are doing good work in Paris I should mention Apostoli, who is experimenting with electricity. He is an enthusiast, and, perhaps, claims too much for his process sometimes, but he is withal very modest. He read a paper before the Copenhagen Congress which was well received, and since then he has been quietly prosecuting his researches. He treats particularly uterine fibroids and believes that, by using his zinc disc covered with a layer of sculptor's clay, he gets a degree of intensity in his current that gives better results than by any other means. The power of his current is simply immense, and those who have watched him treat his cases and seen the results, claim that he really accomplishes nearly every thing which he claims. Not only does he treat fibroids, lessening hæmorrhage and diminishing the growth, but exudations, etc. He is confident that by his process those conditions from hæmorrhage, etc., for which it is believed, on the Continent, that the uterus should be extirpated, can be cured, and the operation which certainly in our country is not regarded with special favor, can be avoided.

I could not leave Paris without visiting Pasteur's laboratory. During the forenoon I had the opportunity of seeing a large number of people inoculated with the virus, and in the afternoon I was permitted to visit his laboratory and witness the entire process, from chloroforming and inoculating a rabbit, up to the time one perished with all the symptoms of hydrophobia. His dispensary was hardly what I expected to find, as it was smaller than the number of patients demanded. There was not the care taken in preserving the histories of cases which the importance of the work merits, but it is possible that this was attended to in the earlier days of the experiments. Pasteur is here during the forenoon, but does very little except exercise a general supervision over the entire work. There are three rooms in which this is performed, one a large waiting room, another a kind of surgery where dog bites are dressed, and a third room where the inoculations are being carried on. About a hundred each day were being inoculated at the time of my visit, and they were there from almost every inhabitable country upon the globe.

A very brief history of Pasteur's experiments from 1880 up to the present time may not at this time be amiss. Since that time the interest in the subject of hydrophobia has been greatly increased and its study diligently pursued. Many have been the investigations to find the nature of this subtle poison, but probably no one has exhibited the patience and had the means to prosecute his studies equal to Pasteur. He had achieved great renown and placed the world, and particularly his own country, greatly his debtor by his discoveries concerning the diseases of the silk-worm. His conclusions in regard to anthrax and chicken-cholera had also been given to the public, and he seemed to be the man to undertake the investigation of hydrophobia. In 1880 a child died of hydrophobia in the Trousseau hospital, in Paris. Four hours after its death the mucus about the palate was collected and two rabbits were inoculated with it. They died in thirty-six hours, and the saliva from these rabbits produced hydrophobia in others of their kind. It seemed then as if the discovery had already been made that the poisonous element of hydrophobia was in the saliva and nowhere else, but Pasteur proceeded further and examined the saliva with the microscope, and in every case he found a special microbe. He undoubtedly believed at this time that he had found what would in every case produce hydrophobia, but others continued to examine the saliva, and, while the microbe of saliva from hydrophobic patients was easily cultivated, and when injected into rabbits caused disease varying with the quantity injected, it was also found that healthy saliva when injected into the veins also produced symptoms closely resembling hydrophobia. At this time I think the theory of a special microbe was dropped. But the work went on until 1884, and experimenters tried to find whether the poison was in the saliva or the blood, or in the central nervous system, when Pasteur read his paper before the Copenhagen Congress, and stated that it was in the medulla in which the disease is found most concentrated, and since then the virus for commencing the process is taken from this point.

The entire procedure as it is here practiced has been gone over time after time, and is as follows: Take the medulla from a dog which has died of rabies, crush it and put it in sterilized bouillon with all antiseptic precautions, and you are ready to make the first injection into the brain of a rabbit. A rabbit is placed upon the operating table, is chloroformed, the top of its head shaved, an incision made through the integuments, with all antiseptic precautions, the bone trepanned and about two drops of virus is injected under the membranes of the brain. In nine days this rabbit commences to be sick, is paralyzed in its legs, and in thirteen days dies. Rabbits do not seem to suffer at all, and the wounds which are made to inoculate them usually heal in about twenty-four to thirty-six hours. From this rabbit's medulla you inoculate another. This rabbit commences to be sick in fifteen days and dies in eighteen, until finally after nine or ten inoculations the incubation period becomes constant at about eight or nine days, but the virus becomes much stronger than it was when taken from the dog who first died from rabies. That is to say, the virus is so strong that the initial symptoms of hydrophobia commence in about seven days in place of from twenty to forty days as they do when a person is bitten by a mad dog. This, then, is the first principle. Pasteur believes he has demonstrated that the further removing of the virus in a rabbit the stronger it becomes. The medulla from these last rabbits in which the virus is so strong is now taken out, hung in an empty jar, the bottom of which is covered with caustic potash. It is here allowed to remain for twenty days. A little piece of this cord is put into sterilized veal broth and rubbed up, and from one to two drops injected into the rabbit. The second injection is made in the same way, only the medulla is allowed to remain in the jar two days less. The third injection is made after the medulla has been allowed to remain in the jar two days less than the third, and so on for eight or ten injections, when it is believed that the virus which has been introduced into the system by a rabid dog has been neutralized and immunity insured. The second principle which has been demonstrated is, that if the virus be allowed to remain in a glass jar, suspended over caustic potash, for twenty days, it may be injected with impunity into the muscles of human beings, and that the virus when suspended two days shorter time is a little stronger and less poisonous to the individual. In the main what Pasteur tries to demonstrate is that by carrying on a series of successive inoculations he simply renders the poison of hydrophobia inert in the individual. He does not attempt to cure the disease after symptoms have commenced their development. At the time of my visit he had inoculated eighteen hundred people, and there had been subscribed to his institute more than $270,000.

If I am asked what the majority of scientific people on the continent think in regard to Pasteur and his cure for hydrophobia, I should say that the majority of those replying would say, "We are waiting." It is not believed by many that the problem is solved. They have, however, great confidence in

Pasteur, and believe that at the proper time he will announce the truth.

A commission has been appointed from Great Britain to investigate and report, and pending this we must all wait. There is, however, an intense interest in the subject in most of the countries of the old world, and there are those who expect that either by this process, or some other, hydrophobia will become a disease of the past.

There are men in training today who will examine the feasibility of vaccinating or inoculating every dog within certain boundaries, in the hope that immunity from the disease will be established in these animals, and the constant danger to man avoided.

535 Washington Boulevard.

